# Differentiation in native as well as introduced ranges: germination reflects mean and variance in cover of surrounding vegetation

**DOI:** 10.1093/aobpla/ply009

**Published:** 2018-02-03

**Authors:** Tina Heger, Gabriele Nikles, Brooke S Jacobs

**Affiliations:** 1 Technical University of Munich, Restoration Ecology, Emil-Ramann-Str. 6, 85354 Freising, Germany; 2 Berlin-Brandenburg Institute of Advanced Biodiversity Research (BBIB), Altensteinstr. 34, 14195 Berlin, Germany; 3 Department of Plant Sciences, University of California, Davis, One Shields Avenue, Davis, CA 95616, USA

**Keywords:** Bet-hedging, competition, eco-evolutionary experience, facilitation, genetic adaptation, physical and physiological dormancy, preadaptation

## Abstract

Germination, a crucial phase in the life cycle of a plant, can be significantly influenced by competition and facilitation. The aim of this study was to test whether differences in cover of surrounding vegetation can lead to population differentiation in germination behaviour of an annual grassland species, and if so, whether such a differentiation can be found in the native as well as in the introduced range. We used maternal progeny of *Erodium cicutarium* previously propagated under uniform conditions that had been collected in multiple populations in the native and two introduced ranges, in populations representing extremes in terms of mean and variability of the cover of surrounding vegetation. In the first experiment, we tested the effect of germination temperature and mean cover at the source site on germination, and found interlinked effects of these factors. In seeds from one of the introduced ranges (California), we found indication for a 2-fold dormancy, hindering germination at high temperatures even if physical dormancy was broken and water was available. This behaviour was less strong in high cover populations, indicating cross-generational facilitating effects of dense vegetation. In the second experiment, we tested whether spatial variation in cover of surrounding vegetation has an effect on the proportion of dormant seeds. Contrary to our expectations, we found that across source regions, high variance in cover was associated with higher proportions of seeds germinating directly after storage. In all three regions, germination seemed to match the local environment in terms of climate and vegetation cover. We suggest that this is due to a combined effect of introduction of preadapted genotypes and local evolutionary processes.

## Teaser

Germination is a crucial phase in a plant’s life. This holds especially for plants occurring outside of their native ranges, where they may lack adaptation. With two experiments, we found that even in the invaded range of our study species, the germination behaviour matched the climate quite well. Seeds collected in the introduced range in California were able to stay dormant even after the physical barrier to germination had been removed and even if they were offered water. In addition, we found a fine-tuning of germination behaviour to match the surrounding vegetation, which is probably accomplished by rapid evolution.

## Introduction

Germination is a crucial step in the life cycle of plants, and this holds especially true for annuals in situations where resources are limited. Fast germination and a high percentage of germination can help to effectively exploit transient resources (Grime *et al.* 1981). If neighbouring plants are present already during the phase of germination, this can have negative effects on plant fitness (Novoplansky 2009; Novoa and González 2014). It has been shown that percentage germination can be substantially lower if other species are present (Oliveira *et al.* 2014). Consequently, early germination is advantageous in situations involving interspecific competition among seedlings (Burke and Grime 1996). Continuous exposure to a high level of interspecific competition, as found in grassland communities with high cover of grasses and herbs, may thus foster directional selection for early germination (Orrock and Christopher 2010).

It is well-known that different populations of the same species can vary in their germination behaviour (e.g. Fenner and Thompson 2005). Variation in requirements for germination, dormancy breaking mechanisms, germination rates and germination speed within species has been observed in many species. They can be due to genetic differentiation as well as maternal effects (Baskin and Baskin 1998, pp. 181 and 187). Population differentiation with respect to germination responses to gradients in temperature can occur within the native (Schwaegerle and Bazzaz 1987; Postma *et al.* 2016) as well as introduced ranges (Beckmann *et al.* 2011). It has been hypothesized that rapid adaptive changes in germination strategies may contribute to the success of globally distributed invaders (Hierro *et al.* 2009). To our knowledge, it has not been explored, however, whether population differentiation in germination can also be driven by differing levels of cover of the surrounding vegetation, and whether such an evolutionary response (if present) can be rapid enough to also occur within an introduced range.

Factors that can lead to population differentiation are not only the average environmental conditions experienced by the plants, but also the spatial and temporal variability in environmental conditions. In environments where unfavourable conditions occur irregularly, a bet-hedging strategy can evolve (Baskin and Baskin 1998, p. 567). Plants using a bet-hedging strategy produce seeds that differ in their germination timing, while showing no heritable differences. Bet-hedging strategies seem to be ubiquitous (Simons 2011), and can also result from different kinds of environmental variation (Tielboerger *et al.* 2012). Hierro *et al.* (2009), for instance, found delayed germination in pappus seeds of *Centaurea solstitialis* populations that experience greater inter-annual variation in winter precipitation.

Because competition can have negative effects on growth and survival, spatially varying levels of competition may promote a bet-hedging strategy. In environments where it is not predictable whether offspring will face high competition or not, and where therefore adaptive trait differentiation does not happen, it may be advantageous to ‘spread the risk’ and produce some seeds with longer dormancy. An alternative expectation would be that low variability in competition and thus good predictability will lead to directional selection of morphological traits. This may then render it advantageous to wait for a reliable cue instead of germinating rapidly as soon as good conditions for germination are given (cf. Cohen 1967). As far as we know, studies testing whether high or low variability in levels of competition leads to delayed germination are not available yet.

We approached these research questions with two germination experiments using seeds from the native and two introduced ranges of the annual grassland species *Erodium cicutarium*. This species is native to Europe, has been transported to many regions of the world and is now considered an invasive species, e.g., in California (USA) and in Chile (Figueroa *et al.* 2004; Brooks and Berry 2006). Competition has been shown to have transgenerational effects on trait expression in this species (Heger *et al.* 2014; Heger 2016), and the populations are differentiated in response to environmental heterogeneity also in the introduced range (Baythavong and Stanton 2010; Baythavong 2011). In the first experiment, we specifically wanted to know if competition at a source site, estimated as mean cover of surrounding vegetation, has an effect on the germination ability of the populations. We expected that seeds from high cover sites would germinate earlier than those from sites with lower cover (Hypothesis 1). Further, we wanted to test whether the source populations differ in their germination response to temperature, and if so, whether these differences can be related to the region the populations occur in (native range: Germany; introduced range: Chile and California) or mean vegetation cover at the source site. We expected that populations from hot climates would differ in germination percentage and speed under high temperatures from populations from temperate climate, but that these differences would be less pronounced if cover at the source site had been high (Hypothesis 2). With the second experiment, we additionally wanted to know whether across regions, seeds from populations with highly variable vegetation cover have higher proportions of dormant seeds than those from population with low variability in cover (Hypothesis 3).

## Methods

### Study species: *E. cicutarium*, redstem filaree

*Erodium cicutarium* is a winter annual to biennial herb in the Geraniaceae family. It occurs on various soil types from soils rich in lime to lime deficient, and prefers dry conditions (Hegi 1964). It can be found in open vegetation such as sand dunes and nutrient-poor grasslands, but also in dense lawns and ruderal vegetation. The pink flowers are mostly self-pollinated. The long seedpod is shaped like the bill of a stork. When fruits are ripe, dry conditions stimulate a spring mechanism powered by the awns curling up in a spiral, and up to five achenes are dispersed ballistically. If humidity changes, the spiralled awns wind and unwind. This movement can cause self-burial of the achenes in the soil (Evangelista *et al.* 2011).

One seed is contained in each achene, so each fruit may hold up to five seeds. A single plant can produce up to 9900 seeds (Blackshaw and Harker 1998), but this maximum is reached only under optimum conditions, and usually seed production is much lower (own observations). In the fresh fruit, physiological and physical dormancy both hinder germination; later, physical dormancy prevails. Seeds have a hard seed coat, and scarification can break physical dormancy (Baskin and Baskin 1998, p. 376). No study is available on whether mechanical scarification, e.g., by abrasion from soil particles happens under natural conditions (Baskin and Baskin [2000] report this lack of evidence for other species, too). For two closely related species in the genus *Erodium*, however, it has been shown that changes in temperature can induce germination (Rice 1985). In an experiment testing germination ability after dry storage, an increase in germination rate during the duration of the experiment (i.e. 2 years) has been observed (Van Assche and Vandelook 2006). The seed coat impermeability in *E. cicutarium* thus can be removed by mechanical scarification, by dry storage or by temperatures fluctuating between cool and hot. When either of these factors renders the seed coat permeable, seeds germinate as soon as conditions are favourable (Rice 1987; Van Assche and Vandelook 2006). In a comparison of the seed bank persistence of 70 dicotyledons in Britain, however, *E. cicutarium* was one of the species with the most persistent seed bank. In the fifth year after sowing, still 4 % of the sown seeds germinated (Roberts 1986). Baskin and Baskin (1998, p. 376) report the optimum temperature for germination as 15 °C/20 °C (night/day).

The species is native to Europe and invasive in many parts of the world. For this study, we collected maternal seed families in the native range (Bavaria, Germany) and in two introduced ranges (California, USA, and Chile). Pollen analysis indicates that *E. cicutarium* has been introduced to California prior to the first settlements of Spanish missionaries, and that it was already well established in the 1750s (Mensing and Byrne 1998). In Chile, the species has most probably been introduced more recently (i.e. during the 19th century).

### Experiment 1: does vegetation cover at source site influence germination ability and response to temperature?

#### Seed sources.

We chose seed collection sites differing in vegetation cover **[seeSupporting Information—Table S1]** and made sure that the sites had not been subject to recent changes in land use. At each collection site, we randomly selected mature seeds from up to 20 individuals and stored them in paper envelopes. In Germany and California, percentage of cover of the surrounding vegetation was estimated in three 50 × 50 cm plots per collection site, each centred on an *E. cicutarium* plant (cf. Heger 2016). In Chile, percentage of vegetation cover was estimated in 15 cm radiuses surrounding each sampled individual. We used these data to calculate means and variances of vegetation cover per population. With an ANOVA (function ‘aov’ in R version 3.1.2; R Core Team 2014) we tested whether seed sources classified as ‘high cover’ differed systematically in their mean daily temperatures from those classified as ‘low cover’, but neither the main effect of cover at source site nor its interaction with source region were significant.

We randomly selected two brown, filled seeds from each field collected maternal family and propagated them in the glasshouse under uniform conditions. Upon flowering, individuals were allowed to produce self-fertilized seeds. After 4 months, we harvested all ripe seeds. Seeds from collection sites in Germany and California were propagated at Gewächshauslaborzentrum Dürnast, Technical University of Munich, those collected in Chile were propagated at the glasshouse facilities of the University of California-Davis. All seeds were stored in paper envelopes at room temperature and under dry conditions after harvesting until the beginning of the experiment.

#### Experimental setup.

In the native range, a germination peak in autumn can be observed in *E. cicutarium* (Poschlod *et al.* 2003). In the summer dry regions in California and Chile, germination usually also starts after the first rains in autumn (Rice 1985 for two close relatives in California). We retrieved daily temperature maxima and minima during autumn (i.e. September through November for Germany and California, and April through June for Chile) from climate stations as close as possible to the sampling sites (mappedplanet.de; data from IPCC, 1960 to 1990). We calculated means for every region and used these to programme a climate chamber (Mueve TK 252; day/night cycle of 12 h without light). Programmed temperature treatments were 13.0 °C/4.6 °C for German autumn, 24.2 °C/8.4 °C for Californian autumn and 16.2 °C/6.6 °C for Chilean autumn.

From each of the three source regions, we selected those five collection sites with the highest and those five sites with the lowest mean cover of vegetation surrounding *E. cicutarium***[seeSupporting Information—Table S1]**. Ten maternal seed families were randomly selected per population. Within each of the six groups of vegetation cover at source site (two levels: high and low) crossed with source region (three levels), 360 seeds were drawn at random across all selected populations and maternal families. We selected only brown, filled seeds. The seeds were pooled per group. For every combination of cover × source region × germination temperature, we loaded six petri dishes with 20 seeds each. We spread the seeds homogeneously on filter paper within each petri dish. In the later analyses, the data for the 120 seeds were accumulated; petri dish was included as random factor. Prior to this, all seeds were physically scarified by placing 10 seeds at a time between two small sheets of sandpaper (Klingspor PL 31 grain size 150). Using light pressure, the top sheet was moved 15 s back and forth and 15 s from left to right.

To make sure all seeds had access to approximately the same amount of water during the experiment and to prevent moulding, we prepared solutions of polyethylene glycol 6000 (PEG-6000) and created a water potential within the optimum range for *E. cicutarium* according to Blackshaw (1992)**[seeSupporting Information—Table S2]**. We pipetted 4 mL of this solution into each petri dish, covered it with parafilm and placed it at a random position into the preheated germination chamber. Due to space limitations we had to run one temperature treatment after the other. Every other day, we recorded and removed germinated seeds. A seed was classified as germinated if the radicle was visible. After 2 weeks, we terminated the experiment. Seeds were regarded as viable but dormant if they were still brown and filled.

#### Statistical analysis.

For analysing data of Experiment 1 we used a time-to-event analysis as suggested by McNair *et al.* (2012). In time-to-event analyses, the fate of single seeds is followed instead of looking at cumulative germination or specific qualities of the germination process of a cohort of seeds (e.g. percentage germinated seeds in a petri dish). To characterize and visualize the temporal pattern of germination, we computed life table estimates of survivor functions for each of the 18 groups of seeds defined by the combination of treatment factors (3 × 3 × 2 for the factors germination temperature, seed source region and vegetation cover at source site). This was done using the ‘lifetab’ function from the ‘KMsurve’ package (Klein *et al.* 2012) in R version 3.1.2 (R Core Team 2014). In the case of germination data, the survival function helps to estimate the probability for a seed to not germinate (i.e. to ‘survive’) during the upcoming time interval.

To statistically compare germination among treatment groups, and to assess the significance of the three covariates germination temperature, seed source region and vegetation cover at source site we used Cox models, following the procedure proposed in McNair *et al.* (2012). Before building the models, we checked the proportional hazards assumption for each covariate based Kaplan–Meier survivor functions (function ‘survfit’ in package ‘survival’; Therneau 2015), to make sure the data meet the requirements for applying these kinds of models. Covariates were then checked for multicolinearity using the variance inflation factor (function ‘vif’ in package ‘HH’; Heiberg 2016). Next, all covariates meeting these requirements were transformed into dummy variables, each named after the factor level which is re-coded as ‘1’ **[see****Supporting Information—Table S3****]**. Cox models were built starting with one covariate (re-coded as dummy) plus the frailty term using the function ‘coxph’ from the package ‘survival’. We then used a forward selection procedure based on the Akaike Information Criterion (function ‘AIC’; R Core Team 2014) to build the final model.

### Experiment 2: do more seeds from populations with highly variable cover of surrounding vegetation stay dormant?

#### Seed sources.

For Experiment 2, we again used seeds originating from the three regions Germany, California and Chile. For information on seed collection methods for German and Chilean origins see Experiment 1. The Californian seed families were collected at 24 sites, using the same protocol for sampling and vegetation surveying as described above for the Chilean sources. All families were propagated at the glasshouse facilities of the University of California-Davis under uniform conditions using the protocol described above.

#### Experimental setup.

For each source site, the variance of percentage cover was calculated from the single cover estimates. We selected the two populations with the highest and those two with the lowest variance in percentage cover **[see****Supporting Information—Table S4****]**. Per population, we randomly selected up to 10 maternal families (118 families in total), and up to eight brown and filled seeds from every family. We equipped eight racks usually used for molecular genetic analyses (e.g. PCR) each with 118 Eppendorf tubes (1.5 mL). Every tube was filled with cotton wool, and tubes were watered with distilled water. We assigned individual seeds randomly to tubes and placed them on top of the wool without prior scarification. We made sure that every maternal family was represented in each rack so that every rack was a replicate (for some families this was not possible because not enough seeds were available **[see****Supporting Information—Table S4****]**). Racks were loosely covered with parafilm.

#### Phase 1—germination after dry storage.

To test for germination directly after the period of dry storage, we placed racks containing the non-scarified seeds in a germination chamber set to day/night cycles of 12 h (always dark) with temperatures at 41 °C/21 °C. Similar temperatures have been observed under field conditions in California in August (Rice 1985). Under these conditions, we expected those seeds to germinate for which the impermeability of the seed coat had been broken by the previous storage, and which are not restrained by high temperature. We recorded germination every other day and removed germinated seeds. When germination had slowed, we switched to a weekly census. At every census we randomly exchanged the position of the racks in the chamber, and watered seeds with distilled water if necessary. Moulded seeds were cleaned with distilled water and placed back in the tube containing fresh cotton wool. If this was not possible because seeds were decayed they were removed and the loss was recorded. When germination had stopped, seeds were still stored in the chamber with the same temperature cycles.

After 148 days (21 weeks), we removed the racks from the chambers and stored them in paper bags at room temperature for 5 months. Brown, filled seeds were regarded as viable and left in the tubes, the others were removed and recorded as dead. To check whether after this further period of dry storage more seeds would germinate we placed the racks again into the chamber for 17 days, using the same temperature cycle.

#### Phase 2—germination after dry-hot treatment.

Next, we wanted to check whether for the remaining seeds, exposure to dry and hot conditions with fluctuating temperature would break dormancy as suggested in Rice (1985). We stopped watering the tubes, removed the parafilm, and changed the temperature cycle to 45 °C/25 °C. We left the seeds under these conditions for 110 days. After this period we surveyed the seeds once more, watered the tubes containing seeds classified as viable with distilled water and placed the racks into the chamber set to the optimum germination conditions according to Blackshaw (1992), i.e. a 12 h cycle of 21 °C/4 °C. We checked every 2 to 4 days for germination. After 20 days, we surveyed again and removed non-viable seeds.

#### Phase 3—germination after scarification.

As a next step, we scarified the remaining seeds by cutting the tip with a razor blade. The aim of this treatment was to find out whether for some seeds, neither dry cool storage nor storage under hot fluctuating temperatures had terminated the impermeability of the seed coat. We watered the seeds and placed the racks back into the chamber using the same optimum conditions as described in the previous paragraph. We surveyed again every 2 to 4 days for 35 days.

#### Statistical analysis.

For the analysis of data from Experiment 2, we wanted to know if seed sources differ concerning which mechanisms break their physical dormancy. We therefore did not focus on the germination percentage over time observed in the different seed sources as in the previous analysis for Experiment 1. Instead, we assigned each seed to a category depending on whether it germinated during one of the three experimental phases or not. Many seeds moulded during the experiment, which made us establish an additional category for these events. Our response variable thus had the five categories ‘dry storage’, ‘dry-hot’, ‘scarification’, ‘moulded’ and ‘no germination’. Some seeds were lost during the experiment and were excluded from the data set.

To test for the effects of seed source region (factor with the three levels Germany, California and Chile), variance of cover at the source site (continuous variable) and their interaction on this categorical response variable, we performed a multinomial logistic regression using the function ‘multinom’ in the package ‘nnet’ (Venables and Ripley 2002). We ordered the levels of the response variable to make ‘dry storage’ the baseline category. We calculated *P*-values based on Wald ratios using the function ‘pchisq’. Overall significance of the effect variables was checked using the function ‘Anova’ from the package ‘car’ (Fox and Weisberg 2011).

## Results

### Effects of temperature and mean vegetation cover at source site on germination

**Figure 1.**
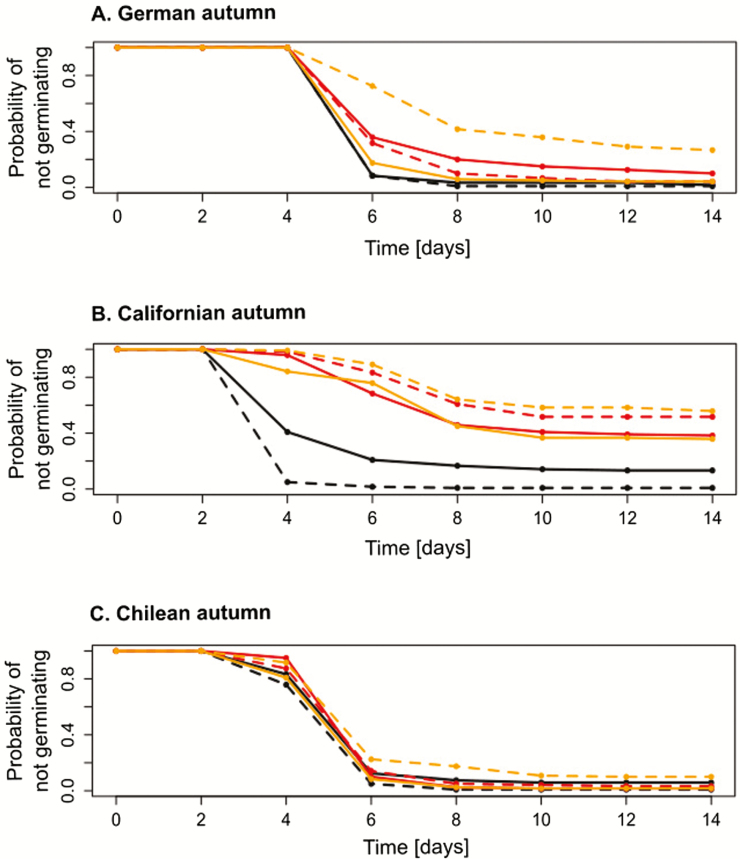
Life table estimates of survivor functions for Experiment 1 for germination temperatures simulating German (A), Californian (B) and Chilean (C) autumn. The probability of not germinating is plotted against time. Colours are coding the seed source regions (black: Germany; red: California; orange: Chile). Solid lines summarize data for seeds from high cover source sites; dashed lines those from low cover sites. For every group, data from 120 seeds have been accumulated.

The results of the final Cox model (Table 1) together with the plotted life table estimates of survivor functions (Fig. 1) show that in the first experiment, the germination temperature had a significant influence on germination of all seeds. Temperatures simulating German autumn slowed the germination process compared to the other two temperatures. Under temperatures simulating Chilean autumn, all groups of seeds showed similar germination behaviour, and nearly all of the seeds in this treatment had germinated by the end of the experiment. Germination patterns under temperatures simulating Californian autumn were significantly different from those under the other two regimes (Table 1; Fig. 1B). The graphs in Fig. 1B suggest that under these conditions, seeds from German sources germinated earlier and to a higher percentage than seeds from the other regions. According to the results of the final Cox model (Table 1), Californian seeds, in particular, had a comparably low chance of germinating under these conditions at any given point in time. This is indicated by the low value of exp(coef) for the interaction of ‘temperature California’ and ‘source region California’ (cf. McNair *et al.* 2012). However, Californian seeds originating from a population with high cover of surrounding vegetation had, under Californian temperatures and compared to seeds from low cover sites, a significantly higher probability for germination at any given time during the experiment (high value of exp(coef) in Table 1). The coefficient was negative, opposed to this, for the significant interaction of temperatures simulating Californian autumn and high cover at source site, indicating a low probability of germination for this treatment combination (Table 1).

**Table 1.** Summary table of the final Cox model for the data from Experiment 1. SE denotes the standard error. Covariates are dummy variables derived from the original categorical variables germination temperature (levels German, Californian, Chilean autumn), seed source region (Germany, California, Chile) and cover at source site (high, low). The dummy variables are coded as 1 if the factor level is the one given in the name of the covariate (e.g. ‘Temperature California’ compares germination of seeds under Californian temperature to those under the other two temperatures **[see****Supporting Information—Table S3****]**). Significant results (*P* < 0.05) are highlighted with bold font.

Covariate	Coefficient	exp(coef)	SE of coef	Chisq	*P*
**Temperature California**	**1.427**	**4.167**	**0.2717**	**27.59**	**<0.001**
Source region California	−0.021	0.980	0.2947	0.00	0.940
Cover at source high	0.197	1.217	0.2387	0.68	0.410
**Temperature California: source region California**	−**3.064**	**0.047**	**0.4963**	**38.12**	**<0.001**
**Temperature California: cover at source high**	−**1.788**	**0.167**	**0.3948**	**20.51**	**<0.001**
Source region California: cover at source high	−0.242	0.785	0.4157	0.34	0.560
**Temperature California: source region California: cover at source high**	**2.346**	**10.442**	**0.7083**	**10.97**	**<0.001**
**Frailty (petri dish**)				**690.29**	**<0.001**

The final Cox model did not reveal a significant influence of vegetation cover at the source sites on germination in general. Nevertheless, the survivor functions indicate some differences among these seed sources. Chilean seeds from high cover source sites germinated earlier and to a higher percentage than those from the Chilean low cover source sites; especially under temperatures of German autumn. German seeds from higher cover source sites germinated later and to a lower percentage under Californian temperatures than German seeds from low cover sites.

### Significant effects of variation in cover at source site on germination

The multinomial logistic regression revealed highly significant effects of source region, variance of cover of surrounding vegetation at source site and the interaction of both (all *P* < 0.001). Whether seeds germinated after storage, after dry-hot treatment or after scarification was significantly influenced by the region the seeds originated from, the degree of spatial variation in the cover of surrounding vegetation at the source site and their interaction.

**Figure 2.**
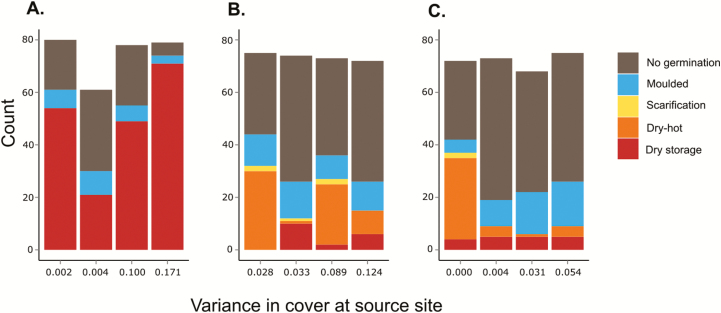
Cumulated number of seeds that germinated during one of the three phases of the experiment, or that moulded or did not germinate, given separately for (A) German, (B) Californian and (C) Chilean seed sources. Each bar represents one source population (cf. **Supporting Information—Table S4**). The *x*-axis gives the variance of cover estimated at the source sites (note: for practical reasons variance is graphed as levels and axes do not give continuous values).

Seeds originating from German populations showed the highest overall proportions of germination (Fig. 2). Those German seeds that germinated did so directly after storage; neither the dry-hot treatment nor scarification with a razor blade caused additional germination events. Compared to seeds from Germany, seeds from California and Chile had a much higher probability to germinate after dry-hot treatment or scarification (cf. high positive coefficients for these categories in Table 2). Across source regions, a more heterogeneous cover at the source site was connected to a higher probability for seeds to germinate after dry storage instead of after dry-hot treatment (high negative coefficient in Table 2). This was true especially for seeds from Chile. Looking at the interaction between origin California and variance in cover, the pattern was reversed: for every unit of increase in variance of cover at the source site, the probability to germinate after dry-hot treatment (as compared to germination after dry storage) increased significantly (log odds increased by 18.9; Table 2).

**Table 2.** Results of a multinomial regression with reference category ‘spontaneous’. Coefficients are given in bold where *P*-values calculated from Wald ratios^1^ indicated significance (*P* < 0.05). The coefficients give an estimate for the log odds for each category in comparison to the baseline, i.e. ‘spontaneous’. For instance, a one-unit increase in variance in cover is associated with a decrease in the log odds of germinating after dry-hot treatment versus spontaneously of 21.36. ^1^R code used to calculate *P*-values: pchisq(summary.MNL$Wald.ratios^2, 1, low = F).

	(Intercept)	Source region California	Source region Chile	Variance of cover at source site	Source region California × variance of cover	Source region Chile × variance of cover
Dry-hot	−27.6428	**29.0628**	**29.2283**	−**21.3641**	**18.8987**	−**38.3071**
Scarification	−19.7016	**19.0287**	**18.7088**	−81.8308	71.9138	−151.2599
Moulded	−1.4749	**2.5771**	**2.1557**	−**8.7310**	6.3293	17.5775
No germination	−0.2848	**2.3961**	**2.6281**	−**9.8595**	11.1567	5.8745

For all seed sources, seeds that germinated after dry storage did so during the first 36 days, afterwards (even after further storage for 5 months) no additional germination occurred. In this experiment, a relatively high proportion of seeds moulded (13.6 %). A chi-square test (function ‘chisq.test’) revealed a significant difference in proportions of moulded seeds across regions (*P* = 0.006), with German seeds showing the lowest percentage of moulding (8.4 %). For seeds from California and Chile, the probability to mould was even higher than the probability to germinate directly after storage (Table 2).

## Discussion

### Interlinked effects of temperature, source region and mean vegetation cover at source site

The results from Experiment 1 show that mean cover of surrounding vegetation at the source site is linked to differences in germination, but only in interaction with temperature and source region. We found early germination in populations from high cover at source site (see Hypothesis 1) for Chilean seeds grown under temperatures simulating German autumn, but not for the other sources or temperatures. High cover of surrounding vegetation thus does not seem to impose a clear enough selective pressure to lead to early germination across regions and temperatures.

Temperature overall had a strong influence on germination. In the intermediate temperature regime created to simulate Chilean autumn, all populations were similar in terms of germination velocity and percentage of germinated seeds. However, in the temperature regime with the highest temperatures (i.e. the one simulating Californian autumn), we observed pronounced differences in germination. For seeds from the cool German climate, high temperatures induced early germination. Seeds originating from California (and according to the survivor functions also those from Chile) showed reduced and retarded germination under these high temperatures. Although the seeds had been scarified, and although water was available, many seeds originating from California and Chile did not germinate.

Baskin and Baskin (1998, pp. 305 and 307) report rapid germination of *E. cicutarium* seeds following rain in the field, and identify scarification as the main dormancy breaking mechanism (p. 376). For seeds originating from Germany, this seems to be true, but not for the other two source areas. Seeds originating from California (and probably those from Chile) instead seem to have an enduring combinatory dormancy: in addition to physical dormancy, which is due to the hard seed coat and can be broken by scarification, physiological dormancy is hindering germination even after dry storage and even if the seed coat is permeable and water is available. For winter annuals with relatively weak physiological dormancy, it has been observed that high summer temperatures gradually break dormancy. If such seeds are exposed to high temperatures, they first gain the ability to germinate at low temperatures (e.g. 6 °C/15 °C), and only with additional loss of dormancy are they able to also germinate at higher temperatures (e.g. 15 °C/25 °C; Baskin and Baskin 1998, p. 58). A combination of physical and physiological dormancy has been reported for *Geranium carolinianum* (Baskin and Baskin 1998, p. 123) and *G. robertianum* (Vandelook and Van Assche 2010) which, along with *E. cicutarium*, are members of the Geraniaceae. Based on experiments done with seeds collected along roadsides in Belgium, Van Assche and Vandelook (2006) propose that both forms of dormancy also exist in seeds of *E. cicutarium*, and that both at the same time can be broken by dry storage leading to high germination percentages at low temperatures in autumn. It is very interesting that in our study, seeds originating from California remained dormant in high fractions, although they had been stored for 12 months, exactly like the German seeds (only Chilean seeds had been stored for a longer time period). For the Californian seeds, this period of dry storage did not break the physiological dormancy.

Kudoh *et al.* (2007) observed suppressed germination under higher temperature regimes in invasive populations of *Cardamine hirsuta*. Temperature-dependent dormancy in this species seems to be mediated by abscisic acid (ABA). Native populations did not consistently show this behaviour; patterns of temperature effects on germination in the native populations were quite variable. Kudoh *et al.* (2007) suggest that suppressed germination at high temperature is a mechanism to assure germination in autumn, and thus to reinforce a winter annual life cycle. This suggestion seems reasonable also for our study system. Availability of water resulting from early rainfall events may not be a good enough predictor for good growing conditions in Mediterranean climates, because short periods of rain may still be followed by hot and dry periods. Only the combination of available water and cooler temperatures may be a reliable cue for a grassland annual in California and Chile assuring high survival rates of germinated seedlings. Further research is needed to clarify the mechanisms underlying the observed suppressed germination in these populations.

Altered germination strategies in the introduced range have been observed in other species (Beckmann *et al.* 2011). However, based on our results, it is not clear whether *E. cicutarium* dormancy strategies evolved in the new range, or whether they have been carried over from a different part of the native range. To be better able to decide on this question, the experiment needs to be repeated with seeds from populations in the native Mediterranean region, especially because the likely region of origin of the Californian and Chilean populations is Spain.

Seeds originating from high cover sources in California (and according to the life table estimates, also those from Chile) germinated to a higher percentage and also faster than those from low cover sources in these regions. The high cover of surrounding vegetation may have had a facilitative effect, attenuating the stressful climatic conditions in California and Chile. This observation is in line with Hypothesis 2. As low germination percentages can be connected to the cost of lower overall abundances, selection may have favoured higher fixed rates of germination in populations where such facilitative effects can be expected. The faster germination may have helped to lessen the negative effects of high cover of neighbours in later life stages.

German seed sources grown under the hot Californian temperatures showed a pattern very different from those of the other two source regions. Here, seeds from high cover sources germinated later than those from low cover sources, and a slightly higher proportion of seeds stayed dormant. Overall, German seed sources germinated earlier under Californian temperatures than under the cooler temperature regimes. The warmer temperatures seem to favour germination in these sources. In German sites, warm temperatures are not necessarily connected to drought, and there probably was no selective pressure inducing a forced dormancy under high temperatures as in California and Chile. In the temperate climate, a strict binding of germination to autumn temperatures may not be necessary.

### Higher variability in vegetation cover at the source site is connected to higher probability for germination directly after storage

Jimenez *et al.* (2016) suggest, based on low germination percentages and high coefficients of variation in seed size in Chile, that *E. cicutarium* is characterized by a diversified bet-hedging strategy. Based on our data, it is not possible to test this suggestion for the species in general. The relatively high proportions of seeds that did not germinate after physical dormancy was broken (see Fig. 2), however, may be seen as indication for the existence of a bet-hedging strategy in this species. In any case, according to the results of Experiment 2, there is no indication that high spatial variability in vegetation cover induces delayed germination in a high proportion of seeds as would be expected for a bet-hedging strategy. Instead, across source regions, high variance in vegetation cover at the source site is associated with higher probability for germination after dry storage, and thus lower numbers of dormant seeds. A potential explanation for this observation is that if the future level of competition is unpredictable, it may be advantageous to germinate as soon as current temperature and water availability allow for it, because high levels of competition could occur in which case fast germination could be advantageous. In cases where future levels of competition (and facilitation) are more predictable, on the other hand, it may be advantageous to wait for environmental cues like dry-hot scarification, to assure a better timing of the germination event. Because potential negative effects of competitors here occur regularly, they could have triggered adaptive variation in morphological or other traits, making early germination less important. Heger *et al.* (2014) observed lower vegetative biomass production and slower growth rates, and at the same time lower abortion rates of developing seed in plants originating from populations with high vegetation cover.

Looking at seeds that originated in California only, though, this result is reversed: the more variable the vegetation cover, the less seeds germinate directly after dry storage. A possible interpretation is that in California, where we found delayed germination also in response to high temperatures, unpredictability of future cover of neighbours represents an additional stressor also inducing a delay in germination. Follow-up experiments testing for the interaction of high cover and high variability in cover are needed to further clarify the mechanisms causing these observations. Since our experimental phases did not simulate the natural sequence of temperatures in either range, it would be interesting to repeat this experiment in the field, ideally with a higher number of seeds to be better able to mirror the high number of seeds produced by this species.

### Germination behaviour and population differentiation match climatic conditions and vegetation cover in native as well as introduced ranges

Experiment 2 showed that German seeds are able to germinate after 12 months of storage at room temperature, whereas seeds originating from Chile and California showed significantly higher probabilities to germinate after dry-hot scarification compared to after storage. Experiment 1 revealed a 2-fold dormancy in seeds from California and probably Chile, allowing the seeds to stay dormant, even if scarified, until lower temperatures are reached. Taken together, these results suggest that germination behaviour in *E. cicutarium* varies among these three regions in accordance with the respective climatic conditions. In the Mediterranean regions Chile and California, overall higher levels of dormancy, the occurrence of enduring physiological dormancy in addition to physical dormancy and the responsiveness to dry-hot scarification may all serve to fine-tune germination events to the dry hot summer and rainy winter seasons.

It is likely that native Mediterranean populations show similar germination behaviour, and that already the first plants introduced to California profited from this preadaptedness. Alternatively, rapid adaptation of germination behaviour in the new range may have occurred. This has been found for *C. solstitialis* (Hierro *et al.* 2009) as well as for *Achillea millefolium*, *Hieracium pilosella* and *Hypericum perforatum* (Beckmann *et al.* 2011). Leiblein-Wild and Tackenberg (2014) found for *Ambrosia artemisiifolia* that populations in the introduced range matched environmental conditions; this was not found, though, when investigating germination rate and speed (Leiblein-Wild *et al.* 2014). Whether preadaptation or evolution in the novel range have led to the observed patterns in *E. cicutarium* has to be clarified in future studies, e.g., by including populations from different parts of the native range.

While the effect of mean cover of surrounding vegetation on germination behaviour was not consistent across regions, we found an effect of variability of cover that was independent of seed source region. This indicates that in all three ranges, populations are differentiated in terms of their germination behaviour as a consequence of differences among populations in cover of surrounding vegetation. It seems unlikely that this pattern is due to the introduction of several preadapted genotypes followed by sorting processes. It is much more probable that such a behaviour has evolved in each population in each of the ranges independently. Parallel evolution in the native and introduced range has been observed before (Maron *et al.* 2004), but we found no study indicating that such a pattern can arise in response to cover of surrounding vegetation.

In both experiments, seeds from German origins tended to germinate earlier and to higher proportions than Californian and Chilean seeds. This pattern seems to contradict findings in other species that invasive populations have higher germination rates (Beckmann *et al.* 2011). Our experiment, though, was not designed to allow for direct comparisons of native and invasive populations. Native regions have not been replicated; moreover, it is very likely that the populations in California and Chile originate from native populations in Spain and not Germany.

## Conclusions

Our study indicates that the cover of surrounding vegetation experienced in a population can influence germination behaviour across generations. Climate and vegetation as well as other factors present in the ecosystems may influence population differentiation and germination behaviour. Our results, however, indicate that in the study systems, differing levels of vegetation cover have driven population differentiation in germination behaviour. This evolutionary response was rapid enough to also occur within the introduced range. Because we had harvested the seeds used in the experiments from plants grown under uniform, glasshouse conditions, we suppose that maternal effects do not contribute significantly to the patterns we found.

For spatial variation in vegetation cover, we found effects that were independent of source region of the seeds, with higher variability generally being connected to higher fractions of seed germinating after dry storage. Mean cover of surrounding vegetation, though, seems to affect the germination behaviour of populations differently depending on the source region. In seeds from Mediterranean climates, especially from California, we observed delayed germination under hot temperatures even though we had scarified the seeds and water was available, which we interpret as indication for an additional physiological dormancy in these populations. The delay in germination was less strong in populations that had experienced high cover of surrounding vegetation. We suggest that high vegetation cover ameliorates heat and drought, thus making the need for delayed germination less urgent. Under these conditions of potentially strong selective pressures caused by heat and drought, the facilitative effects of neighbours may be more important than competitive effects during later life stages.

The strong differences in dormancy behaviour we found among the populations in the native range and the introduced ranges, with different cues inducing germination and an additional physiological dormancy only in Californian (and maybe also Chilean) populations, may be due either to the introduction of genotypes preadapted to the Mediterranean climates in the introduced range, or to evolutionary changes in the new range. We believe the first option to be more likely. The differentiation we observed in accordance with mean and variance in vegetation cover, opposed to this, we interpret as fine-tuned adjustments to the local environments, which are more likely to have evolved locally in each of the populations.

## Sources of Funding

T.H. received funding by Deutsche Forschungsgemein-schaft (DFG) (grant numbers HE 5893/3-1 and HE 5893/3-2). B.S.J. was supported by a NSF International Research Fellowship Award (grant #0853094). This work was supported by the German Research Foundation (DFG) and the Technical University of Munich (TUM) in the framework of the Open Access Publishing Programme.

## Contributions by the Authors

B.S.J. and T.H. collected the seeds and conceived and designed the experiments; G.N. conducted the experiments; T.H. performed the statistical analyses in the presented version and wrote the text; B.S.J. and G.N. edited the text.

## Conflict of Interest

None declared.

## Supporting Information

The following additional information is available in the online version of this article—

**Table S1**. Gives information on the collection sites for Experiment 1.

**Table S2**. Characterizes the three temperatures treatments applied in Experiment 1.

**Table S3**. Explains the dummy variables used for the statistical analysis of Experiment 1.

**Table S4**. Gives information of the collections sites for Experiment 2.

## Supplementary Material

Supporting Information

## References

[CIT0001] Baskin CC, Baskin JM 1998 Seeds. Ecology, biogeography, and evolution of dormancy and germination. San Diego, CA: Academic Press.

[CIT0002] Baskin JM, Baskin CC 2000 Evolutionary considerations of claims for physical dormancy-break by microbial action and abrasion by soil particles. Seed Science Research 10:409–413.

[CIT0003] Baythavong BS 2011 Linking the spatial scale of environmental variation and the evolution of phenotypic plasticity: selection favors adaptive plasticity in fine-grained environments. The American Naturalist 178:75–87. 10.1086/660281 21670579

[CIT0004] Baythavong BS, Stanton ML 2010 Characterizing selection on phenotypic plasticity in response to natural environmental heterogeneity. Evolution 64:2904–2920. 20649815 10.1111/j.1558-5646.2010.01057.x

[CIT0005] Beckmann M, Bruelheide H, Erfmeier A 2011 Germination responses of three grassland species differ between native and invasive origins. Ecological Research 26:763–771.

[CIT0006] Blackshaw RE 1992 Soil temperature, soil moisture, and seed burial depth effects on Redstem Filaree (*Erodium cicutarium*) emergence. Weed Science 40:204–207.

[CIT0007] Blackshaw RE, Harker KN 1998 Redstem Filaree (*Erodium cicutarium*) development and productivity under noncompetitive conditions. Weed Technology 12:590–594.

[CIT0008] Brooks ML, Berry KH 2006 Dominance and environmental correlates of alien annual plants in the Mojave Desert, USA. Journal of Arid Environments 67:100–124.

[CIT0009] Burke MJW, Grime JP 1996 An experimental study of plant community invasibility. Ecology 77:776–790.

[CIT0010] Cohen D 1967 Optimizing reproduction in a randomly varying environment when a correlation may exist between the conditions at the time a choice has to be made and the subsequent outcome. Journal of Theoretical Biology 16:1–14. 6035758 10.1016/0022-5193(67)90050-1

[CIT0011] Evangelista D, Hotton S, Dumais J 2011 The mechanics of explosive dispersal and self-burial in the seeds of the filaree, *Erodium cicutarium* (Geraniaceae). The Journal of Experimental Biology 214:521–529. 21270299 10.1242/jeb.050567

[CIT0012] Fenner M, Thompson K 2005 The ecology of seeds. Cambridge, UK; New York, NY: Cambridge University Press.

[CIT0013] Figueroa JA, Castro SA, Marquet PA, Jaksic FM 2004 Exotic plant invasions to the Mediterranean region of Chile: causes, history and impacts. Revista Chilena De Historia Natural 77:465–483.

[CIT0014] Fox J, Weisberg S 2011 An {R} companion to applied regression. Thousand Oaks, CA: Sage.

[CIT0015] Grime JP, Mason G, Curtis AV, Rodman J, Band SR 1981 A comparative study of germination characteristics in a local flora. Journal of Ecology 69:1017–1059.

[CIT0016] Heger T 2016 Light availability experienced in the field affects ability of following generations to respond to shading in an annual grassland plant. Journal of Ecology 104:1432–1440.

[CIT0017] Heger T, Jacobs BS, Latimer AM, Kollmann J, Rice KJ 2014 Does experience with competition matter? Effects of source competitive environment on mean and plastic trait expression in *Erodium cicutarium*. Perspectives in Plant Ecology Evolution and Systematics 16:236–246.

[CIT0018] Hegi G 1964 Illustrierte Flora von Mittel-Europa. Mit besonderer Berücksichtigung von Deutschland, Österreich und der Schweiz. Zum Gebrauch in Schulen und zum Selbstunterricht. Jena, Germany: Weissdorn-Verlag.

[CIT0019] Heiberg RM 2016 Statistical analysis and data display: Heiberger and Holland. R package version 3.1-31. http://CRAN.R-project.org/package=HH. (23 June 2016)

[CIT0020] Hierro JL, Eren Ö, Khetsuriani L, Diaconu A, Török K, Montesinos D, Andonian K, Kikodze D, Janoian L, Villarreal D, Estanga-Mollica ME, Callaway RM 2009 Germination responses of an invasive species in native and non-native ranges. Oikos 118:529–538.

[CIT0021] Jimenez MA, Gaxiola A, Armesto JJ, Gonzalez-Browne C, Meserve PL, Kelt DA, Gutierrez JR, Jaksic FM 2016 Bet-hedging strategies of native and exotic annuals promote coexistence in semiarid Chile. Journal of Arid Environments 126:62–67.

[CIT0022] Klein JP, Moeschenberger ML, Yan J 2012 KMsurv. R package version 0.1-5. Original by Klein, Moeschberger and modifications by Jun Yan (2012). http://CRAN.R-project.org/package=KMsurv (24 June 2016).

[CIT0023] Kudoh H, Nakayama M, Lihová J, Marhold K 2007 Does invasion involve alternation of germination requirements? A comparative study between native and introduced strains of an annual Brassicaceae, *Cardamine hirsuta*. Ecological Research 22:869–875.

[CIT0024] Leiblein-Wild MC, Kaviani R, Tackenberg O 2014 Germination and seedling frost tolerance differ between the native and invasive range in common ragweed. Oecologia 174:739–750. 24197990 10.1007/s00442-013-2813-6 PMC3933736

[CIT0025] Leiblein-Wild MC, Tackenberg O 2014 Phenotypic variation of 38 European *Ambrosia artemisiifolia* populations measured in a common garden experiment. Biological Invasions 16:2003–2015.

[CIT0026] Maron JL, Vila M, Bommarco R, Elmendorf S, Beardsley P 2004 Rapid evolution of an invasive plant. Ecological Monographs 74:261–280.

[CIT0027] McNair JN, Sunkara A, Frobish D 2012 How to analyse seed germination data using statistical time-to-event analysis: non-parametric and semi-parametric methods. Seed Science Research 22:77–95.

[CIT0028] Mensing S, Byrne R 1998 Pre-mission invasion of *Erodium cicutarium* in California. Journal of Biogeography 25:757–762.

[CIT0029] Novoa A, González L 2014 Impacts of *Carpobrotus edulis* (L.) N.E.br. on the germination, establishment and survival of native plants: a clue for assessing its competitive strength. PLoS One 9:e107557. 25210924 10.1371/journal.pone.0107557 PMC4161477

[CIT0030] Novoplansky A 2009 Picking battles wisely: plant behaviour under competition. Plant, Cell & Environment 32:726–741. 10.1111/j.1365-3040.2009.01979.x 19389051

[CIT0031] Oliveira G, Clemente A, Nunes A, Correia O 2014 Suitability and limitations of native species for seed mixtures to re-vegetate degraded areas. Applied Vegetation Science 17:726–736.

[CIT0032] Orrock JL, Christopher CC 2010 Density of intraspecific competitors determines the occurrence and benefits of accelerated germination. American Journal of Botany 97:694–699. 21622431 10.3732/ajb.0900051

[CIT0033] Poschlod P, Kleyer M, Jackel A-K, Dannemann A, Tackenberg O 2003 BIOPOP - a database of plant traits and internet application for nature conservation. Folia Geobotanica 38:263–271.

[CIT0034] Postma FM, Lundemo S, Ågren J 2016 Seed dormancy cycling and mortality differ between two locally adapted populations of *Arabidopsis thaliana*. Annals of Botany 117:249–256. 26637384 10.1093/aob/mcv171 PMC4724045

[CIT0035] R Core Team. 2014 R: a language and environment for statistical computing. Vienna, Austria: R Foundation for Statistical Computing http://www.R-project.org/. (30 October 2015)

[CIT0036] Rice KJ 1985 Responses of *Erodium* to varying microsites: the role of germination cueing. Ecology 66:1651–1657.

[CIT0036a] Rice KJ 1987 Evidence for the retention of genetic variation in erodium seed dormancy by variable rainfall. Oecologia 72:589–596. 28312523 10.1007/BF00378987

[CIT0036b] Roberts HA 1986 Seed persistence in soil and seasonal emergence in plant species from different habitats. Journal of Applied Ecology 23:639–656.

[CIT0037] Schwaegerle KE, Bazzaz FA 1987 Differentiation among nine populations of *Phlox*: response to environmental gradients. Ecology 68:54–64.

[CIT0038] Simons AM 2011 Modes of response to environmental change and the elusive empirical evidence for bet hedging. Proceedings of the Royal Society B-Biological Sciences 278:1601–1609. 10.1098/rspb.2011.0176 PMC3081777 21411456

[CIT0039] Therneau T 2015 A package for survival analysis in S. version 2.38. http://CRAN.R-project.org/package=survival (23 June 2016).

[CIT0040] Tielboerger K, Petru M, Lampei C 2012 Bet-hedging germination in annual plants: a sound empirical test of the theoretical foundations. Oikos 121:1860–1868.

[CIT0041] Van Assche JA, Vandelook FEA 2006 Germination ecology of eleven species of *Geraniaceae* and *Malvaceae*, with special reference to the effects of drying seeds. Seed Science Research 16:283–290.

[CIT0042] Vandelook F, Van Assche JA 2010 A combined physical and physiological dormancy controls seasonal seedling emergence of *Geranium robertianum*. Plant Biology 12:765–771. 20701699 10.1111/j.1438-8677.2009.00290.x

[CIT0043] Venables WN, Ripley BD 2002 Modern applied statistics with S, 4th edn. New York, NY: Springer.

