# Supplementary material for: Differentiation in native as well as introduced ranges: germination reflects mean and variance in cover of surrounding vegetation
Source: AoB Plants. 2018 Feb 3;10(1):ply009. doi: 10.1093/aobpla/ply009 (PMC5815068; doi:10.1093/aobpla/ply009)
Supplement: Supporting Information [file ply009_suppl_supporting_information.docx]

**Supporting information**

**Table S1.** Collection sites for the seeds used in Experiment 1 and their classification into high or low cover depending on vegetation cover estimated in the field. Data on percentage cover represent means per population. Maternal families were collected in 2010 (Chile), 2011 and 2012 (Germany and California); propagated seeds were harvested in May 2011 (Chile) and July 2013 (Germany and California).

| **Region** | **Population** | **Latitude** | **Longitude** | **Percen-tage cover** | **Variance of percen-tage cover** | **Estimate of mean cover** |
| --- | --- | --- | --- | --- | --- | --- |
| Bavaria, Germany | Sandharlanden 2 | 48.84278 | 11.79975 | 0.18 | 0.0208 | low |
|  | München, Westkreuz | 48.18079 | 11.43458 | 0.19 | 0.0103 | low |
|  | München, Mallertshofer Holz | 48.26233 | 11.63750 | 0.35 | 0.0075 | low |
|  | Sandharlanden 1 | 48.84403 | 11.79945 | 0.35 | 0.0175 | low |
|  | München, NSG Panzerwiese | 48.22292 | 11.58512 | 0.40 | 0.0020 | low |
|  | Scheuern | 48.81195 | 11.93133 | 0.58 | 0.0078 | high |
|  | Ilmmünster | 48.48810 | 11.51696 | 0.73 | 0.1706 | high |
|  | Erlangen, Exerzierplatz | 49.58214 | 11.03063 | 0.73 | 0.0433 | high |
|  | München, Neuperlach | 48.09523 | 11.66153 | 0.77 | 0.0049 | high |
|  | Nürnberg, Luitpoldhain | 49.43435 | 11.10605 | 0.92 | 0.0037 | high |
| California, USA | McLaughlin Natural Reserve | 38.82834 | -122.34995 | 0.17 | 0.0133 | low |
|  | Stebbins Cold Canyon Reserve | 38.50864 | -122.09625 | 0.27 | 0.0408 | low |
|  | Davis, Putah Creek | 38.52779 | -121.79911 | 0.33 | 0.0208 | low |
|  | McLaughlin Natural Reserve 2 | 38.82577 | -122.35362 | 0.33 | 0.0133 | low |
|  | Sierra Foothills REC | 39.26394 | -121.32458 | 0.43 | 0.0633 | low |
|  | Sierra Foothills REC | 39.26500 | -121.32481 | 0.66 | 0.0100 | high |
|  | McLaughlin Natural Reserve | 38.82519 | -122.35408 | 0.76 | 0.0036 | high |
|  | Hopland REC | 39.03056 | -123.09525 | 0.80 | 0.0075 | high |
|  | Stebbins Cold Canyon Reserve | 38.50911 | -122.09494 | 0.80 | 0.0075 | high |
|  | Davis, Orchard Park | 38.54391 | -121.76749 | 0.99 | 0.0001 | high |
| Chile | Los Choros | -29.29194 | -71.30444 | 0.06 | 0.0088 | low |
|  | Illapel | -31.58899 | -71.43521 | 0.12 | 0.0078 | low |
|  | Vallenar | -28.62880 | -70.76823 | 0.16 | 0.0118 | low |
|  | Carrizal Bajo | -28.16998 | -71.15720 | 0.17 | 0.0522 | low |
|  | Las Campanas | -32.98045 | -71.11864 | 0.19 | 0.0041 | low |
|  | Los Vilos | -31.88000 | -71.49361 | 0.51 | 0.0908 | high |
|  | Rosita's house | -33.90500 | -70.76528 | 0.56 | 0.0164 | high |
|  | Santiago | -33.47871 | -70.51686 | 0.67 | 0.0535 | high |
|  | Fray Jorge | -30.66189 | -71.68274 | 0.81 | 0.0314 | high |
|  | NeoHaus | -29.91393 | -71.27498 | 1.00 | 0.0000 | high |

**Table S2.** Characterization of the three temperature treatments applied in Experiment 1. Periods of day and night were 12h. The last columns give the PEG-6000 concentration, and the resulting water potential (estimates, based on eq.1 in Michel 1983).

| **Temperature treatment** | **Day temp-erature [°C]** | **Night temp-erature [°C]** | **Mean temp-erature [°C]** | **PEG-6000 concentration [g/l]** | **Water potential [MPa]** |
| --- | --- | --- | --- | --- | --- |
| German autumn | 13.0 | 4.6 | 8.8 | 110.75 | -0.20 |
| Californian autumn | 24.2 | 8.4 | 16.3 | 100.15 | -0.16 |
| Chilean autumn | 16.2 | 6.6 | 11.4 | 106.70 | -0.19 |

**References**

Michel BE. 1983. Evaluation of the water potentials of solutions of Polyethylene Glycol 8000 both in absence and presence of other solutes. *Plant Physiology* 72:66-70.

**Table S3:** Re-coding of covariates for statistical analysis in Experiment 1.

| **Covariate** | **Dummy variable** | **Meaning of “1”** | **Meaning of “0”** |
| --- | --- | --- | --- |
| Temperature | Temperature Bavaria | “Bavarian autumn” | Not “Bavaria autumn” |
|  | Temperature California | “Californian autumn” | Not “Californian autumn” |
| Source region | Source region Bavaria | “Bavaria” | Not “Bavaria” |
|  | Source region California | “California” | Not “California” |
| Competition at source | Competition at source high | “high” | “low” |

**Table S4.** Characterization of collection sites for the seeds used in Experiment 2. Columns three and four give the numbers of maternal families and seeds included per population, and column 7 and 8 give data on vegetation cover estimated in the field. Data on percentage cover represent means per population. Maternal families were collected in 2010 (California and Chile) and 2011 (Germany); propagated seeds were harvested in May 2011 (California and Chile) and March 2012 (Germany).

| **Region** | **Population** |  | **Maternal families** | **Seeds** | **Latitude** | **Longitude** | **Percen-tage cover** | **Variance of percen-tage cover** |
| --- | --- | --- | --- | --- | --- | --- | --- | --- |
| Bavaria, Germany | München, NSG Panzerwiese | PW | 10 | 80 | 48.22292 | 11.58512 | 0.40 | 0.0020 |
|  | Nürnberg, Luitpoldhain | NU | 8 | 64 | 49.43435 | 11.10605 | 0.92 | 0.0037 |
|  | Stockensau | SS | 10 | 80 | 48.51813 | 11.19245 | 0.57 | 0.1001 |
|  | Ilmünster | IL | 10 | 80 | 48.48810 | 11.51696 | 0.73 | 0.1706 |
| California, USA | Motte | MO | 10 | 80 | 33.80446 | -117.25867 | 0.63 | 0.0284 |
|  | Sierra Foothills | SF | 10 | 79 | 39.25750 | -121.28890 | 0.72 | 0.0327 |
|  | Kearney | KE | 10 | 78 | 36.60268 | -119.50808 | 0.37 | 0.0893 |
|  | Mc Laughlin | ML | 10 | 74 | 38.82483 | -122.34739 | 0.50 | 0.1240 |
| Chile | NeoHaus | NH | 10 | 79 | -29.91393 | -71.27498 | 1.00 | 0.0000 |
|  | Las Campanas | LC | 10 | 75 | -32.98045 | -71.11864 | 0.19 | 0.0041 |
|  | Fray Jorge | FJ | 10 | 72 | -30.66189 | -71.68274 | 0.81 | 0.0314 |
|  | Santiago | ST | 10 | 78 | -33.47871 | -70.51686 | 0.67 | 0.0535 |
| **Total** |  |  | **118** | **919** |  |  |  |  |
|  |  |  |  |  |  |  |  |  |
